# Unified dosimetry index (UDI): a figure of merit for ranking treatment plans

**DOI:** 10.1120/jacmp.v9i3.2803

**Published:** 2008-06-23

**Authors:** Hilary Akpati, ChangSeon Kim, Bong Kim, Tae Park, Allen Meek

**Affiliations:** ^1^ Department of Radiation Oncology Stony Brook University School of Medicine Stony Brook New York U.S.A.

**Keywords:** Treatment Planning, Dose Coverage, Dose Conformity, Dose Homogeneity, Dose Gradient

## Abstract

We have formulated a unified dosimetry index (UDI) that computes, for any given treatment plan, its deviations in terms of dose coverage, conformity, homogeneity, and dose gradient vis‐à‐vis an ideal plan (which we define as a dosimetry plan of perfect dose coverage, conformity, homogeneity, and step‐wise fall‐off to zero dose outside the planning target volume). In order to validate the UDI scoring system, 21 stereotactic cranial radiosurgery cases were evaluated retrospectively. The cases were planned on the BrainSCAN treatment planning system (BrainLAB, Feldkirchen, Germany) using 6 to 8 non‐coplanar static beams collimated with the micro multi‐leaf collimator (mMLC). We suggest a technique for creating a ranking system that can be utilized for plan evaluation and comparison between multiple plans. Under this system treatment plans are classified as “excellent”, “good”, “average”, or “poor”. The proposed ranking system can be utilized as a general guide for generating an optimal dosimetry plan for external beam radiation therapy.

## I. INTRODUCTION

Treatment plan evaluation tools are necessary for judging how well dosimetry plans meet the preset planning objectives. Traditional methods such as three dimensional dose distributions, and dose volume histogram (DVH), are adequate for qualitative evaluations, but not well suited for efficient comparison of multiple plans. Furthermore, adequate quantitative comparison of dosimetry plans generated from two different treatment planning systems (TPS) usually requires third‐party software that can import data from the two TPS into a common platform. Dosimetry planning objectives typically consist of (a) achieving full uniform dose coverage to the target (i.e., covering close to 100% of the target with the prescribed dose), (b) attaining high target dose conformity, (c) minimizing the volume of normal tissue receiving very high dose, and (d) limiting the dose to critical structures below their tolerance. While direct scoring of the first three objectives is easily obtained, scoring the fourth objective can be quite complex. In principle, if the dose beyond the target volume falls off sharply, then the dose to critical structures close to the target may also be reduced. Therefore, quantifying the dose gradient is an indirect way of scoring the fourth objective mentioned above.

Several indices for evaluating treatment plan dose coverage, conformity and dose gradient in separate scores as well as in combined overall score have been suggested.^1^, ^11^ However none of the indices incorporates dose homogeneity. Whereas homogeneity may not be of high priority in most treatment plans, in cases such as stereotactic radiosurgery (SRS), in which the target may be in very close proximity to a serial critical organ or nerve (e.g., acoustic neuroma), very high hot spots outside the PTV potentially increases the risk of complication. It has been suggested in a publication by Chang et al.^(12)^ that improved tumor dose homogeneity and a staged treatment regimen may improve hearing preservation in acoustic neuroma patients receiving radiosurgery treatment. Furthermore, for intensity modulated radiation therapy (IMRT) treatments there is a higher likelihood for significant positioning error as well as intra‐ and inter‐fraction motions compared to stereotactic radiosurgery treatments. Thus, high dose homogeneity (hot spots outside the target) is particularly undesirable for IMRT, especially if the target is in close proximity to a critical structure, as is often the case.

Our approach is to formulate a unified dosimetry index (UDF) that computes for any given treatment plan its deviations in terms of dose coverage, conformity, homogeneity, and dose gradient vis‐à‐vis an ideal plan. An ideal plan is defined as one with full uniform dose coverage, perfectly conformed to the target, and a step‐wise fall‐off to zero dose outside the target. An overall score that integrates contributions from all four dosimetry components is obtained for each plan. Details of the unified dosimetry index method are presented in section II. Results of retrospective scoring of 21 cranial radiosurgery treatment plans, demonstrating the effectiveness of the UDI system, are presented in section III. In the discussion section we present a general guideline for creating a ranking system that can be used to classify treatment plans as “excellent”, “good”, “average”, or “poor”.

## II. MATERIALS & METHODS

Dose coverage is defined as the fraction of the planning target volume (PTV) receiving the prescribed dose. Dose conformity, on the other hand, is defined as the ratio of the total volume of all tissue receiving the prescribed dose versus the PTV. Whereas dose coverage gives a measure of how well the PTV is covered by the prescribed dose, dose conformity gives a measure of how well the prescribed dose is confined to the PTV. Dose gradient is defined as the ratio of the volume receiving the prescribed dose and the volume receiving half the prescribed dose. The dose conformity and dose gradient components are defined such that high values are translated as good conformity and high dose gradient (fast dose fall‐off), whereas low scores indicate poor conformity and low dose gradient, respectively. On the other hand, dose homogeneity is defined as the ratio of maximum dose to prescribed dose. Therefore a high dose homogeneity value represents hot spots in and around the planning target volume. However, the definition of dose homogeneity can be restricted to pertain only to hot spots outside the PTV, consistent with the International Commission of Radiation Units and Measurements (ICRU),^13^, ^14^ so that a high homogeneity score (i.e., values much greater than 1.0) represents a “poor” plan, whereas a lower score (values closer to 1.0) would be associated with a “good” plan. Comparing the dosimetry components we see that both dose conformity and dose gradient score “good” plans in an opposite sense as the dose homogeneity component. This presents a challenge in terms of creating an index that integrates either dose conformity or dose gradient components (or both) with homogeneity, into a single scoring system.

The UDI formulation is based on a mathematical logic that scores the overall deviation of a treatment plan vis‐à‐vis an “ideal” plan (as defined above). The unified dosimetry index is defined as follows:

(1) $$UDI=\left\{\prod_{k=1}^{4}W_{k}\cdot \{|1.0-DI_{k}|+0.1\}\right\}\times 10^{4}$$

where

(2) $$DI_{1}\equiv C(Coverage\;Index)=\frac{PTV_{PI}}{PTV}$$

(3) $$DI_{2}\equiv CF(Conformity\;Index)=\frac{DV_{PI}}{PTV}$$

(4) $$DI_{3}\equiv HI(Homogeneity\;Index)=\frac{D_{max}}{D_{PI}}$$

(5) $$DI_{4}\equiv DG(Dose\;Gradient\;Index)=\frac{DV_{PI}}{DV_{HPI}}$$

where PTV is the planning target volume; ${\text{PTV}}_{\text{PI}}=\text{planning target volume receiving the prescribed isodose}\;(\text{PI})$; ${\text{DV}}_{\text{PI}}=\text{dose volume of the prescribed isodose}$; ${\text{DV}}_{\text{HPI}}=\text{dose volume of half the prescribed isodose}$; $D_{\text{Max}}=\text{maximum dose at any point 2 mm beyond the PTV}$; and $D_{\text{PI}}=\text{dose value of the prescribed isodose}$. HI=1, if $D_{\text{Max}}<D_{\text{PI}}$. $W_{k}$ denotes weighting factors that reflect the relative importance of the four components. The weighting factors should be restricted such that $W_{1}\times W_{2}\times W_{3}\times W_{4}=1.0$ (in order to prevent inflation or deflation of the UDI score). The composite index, UDI, compounds the contributions from all four components into one single score as defined in equation (1). It may also be desirable in some situations to analyze each individual component of UDI separately. All four components are expressed in terms of the same generic function, UDI(X), which is defined as follows:

(6) UDI(X)={|1.0−X|+0.1}×10

where X={C, CF, HI, or DG}, defined in equations (2), (5) above, is substituted for coverage, conformity, homogeneity, and dose gradient indices, respectively. All four components, UDI(C), UDI(CF), UDI(HI) and UDI(DG), have the same basic form given in equation (6). This ensures that the deviation contribution from each component (with respect to an “ideal” plan) is positive and normalized such that for an “ideal” plan the composite UDI score equals 1.0. In other words, for an “ideal” plan, the following conditions hold:

(7a) C=1.0;HI=1.0;CF=1.0;DG=1.0

(7b) UDI=UDI(C)×UDI(CF)UDI(HI)×UDI(DG)=1.0

**Figure 1:**
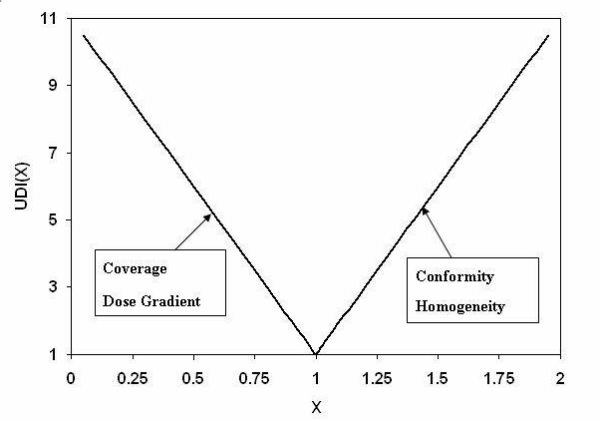
A plot of UDI component generic function, UDI(X). All four components are expressed in terms of the UDI(X) function given in equation (6). Here X can be coverage, dose gradient, conformity, or homogeneity. The dose coverage and dose gradient components are on the negative slope, whereas conformity and homogeneity components are on the positive slope of the function.

For an actual physically realizable dosimetry plan the composite UDI score is always greater than 1.0. The generic form of the UDI components, UDI(X), given in equation (6), is essentially a V‐shaped function centered at an X‐value of 1.0. A plot of UDI(X) is shown in Fig. 1. The dose coverage and dose gradient components, UDI(C) and UDI(DG), are both on the negative slope. This stems from the definitions of coverage and dose gradient given in equations (2) and (5), respectively. On the other hand, the dose conformity and homogeneity components, UDI(CF) and UDI(H), are both on the positive slope (which also stems from definitions of conformity and homogeneity given in equations (3) and (4), respectively).

To test the UDI scoring index, a retrospective evaluation of actual dosimetry plans used for treatment of 21 cranial radiosurgery cases, randomly selected from a pool accumulated over a period of two years, is presented (see Table 1). The only selection criterion is that all cases considered for the study had to have a single isocenter. This ensures that each isocenter dose distribution is devoid of dose contributions from an adjoining isocenter. The dosimetry plans for all 21 cases were obtained using the BrainScan planning system (BrainLAB, Feldkirchen, Germany). 3D‐conformal multiple non‐coplanar static beams (6 to 8) were utilized. The beams were collimated with the micro multi‐leaf collimator (mMLC). The BrainLab m3 model (with 3 mm projected leaf width at isocenter) is utilized.

**Table 1:** Twenty‐one stereotactic radiosurgery cases utilized in the study. The treatment plans were scored retrospectively for the purpose of validation of the UDI scoring system, and in order to establish benchmarks. Notations for the critical organs are as follows: BS=Brain Stem; P=Pituitary; OC=Optic Chiasm; ON=Optic Nerves; E=Eyes; NA=No applicable critical organs.

*Case*	*PTV (cc)*	*Dimensions (cmxcmxcm)*	*Location*	*Critical Organs*	*UDI*
1	2.73	2.0×1.9×1.4	LT Occipital Lobe	NA	255
2	3.13	1.6×1.7×1.5	RT Frontal Lobe	NA	223
3	1.10	0.9×1.3×1.1	RT Cerebellum	BS	320
4	4.21	2.3×3.3×2.4	LT Parietal Lobe	BS	59
5	2.03	1.0×0.8×0.9	RT Frontal Lobe	NA	188
6	1.22	2.0×2.4×1.9	RT Parietal Lobe	BS	78
7	1.11	1.1×1.3×1.1	RT Temporal Lobe	BS, P, OC	391
8	3.60	1.8×1.9×3.0	Occipital Lobe	BS	304
9	13.30	2.3×3.9×2.2	LT Frontal Lobe	OC, BS	76
10	9.38	3.0×2.0×1.3	LT Occipital Lobe	OC, P, ON	501
11	17.90	2.4×1.7×2.8	LT Frontal Lobe	BS	573
12	7.97	2.5×2.5×1.8	LT Occipital Lobe	BS	380
13	2.61	1.6×1.7×1.6	LT Parietal Lobe	BS, P, OC	115
14	1.27	0.6×1.8×0.8	RT Parietal Lobe	BS, ON	427
15	5.79	2.4×2.2×2.1	LT Pons	BS, P, OC	153
16	0.33	0.8×0.8×1.0	LT Cerebellum	BS	242
17	0.07	0.6×0.5×4.0	RT Frontal Lobe	E, ON, OC	280
18	5.13	2.0×2.5×1.9	RT Frontal Lobe	NA	357
19	11.06	2.3×3.3×2.3	RT Superior Parietal	NA	120
20	3.85	1.8×1.7×1.9	LT Cerebellum	BS	183
21	12.75	2.9×2.7×2.6	LT Temporal Lobe	BS, OC	140

## III. RESULTS

**Figure 2:**
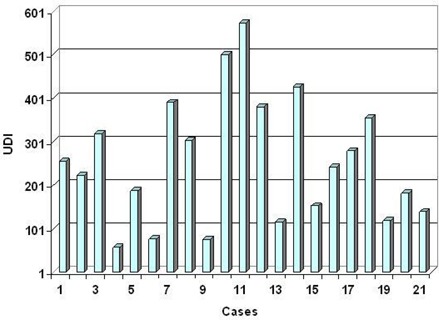
A histogram plot of unified dosimetry index (UDI) for 21 radiosurgery cases. The dosimetry plans were obtained using 3D‐conformal non‐coplanar static beams collimated with BrainLab micro multi‐leaf collimator (mMLC) system.

**Figure 3:**
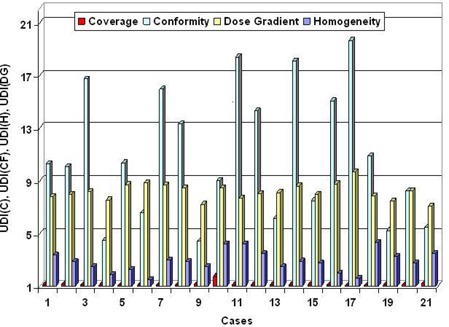
A histogram plot of dosimetry objectives that comprise the four UDI components expressed in terms of equation 1. UDI(C), UDI(CF), UDI(HI), and UDI(DG) denote coverage, conformity, homogeneity and dose gradient, respectively. Conformity and dose gradient are the two dominant components. The homogeneity component is also significant. The coverage component is approximately 1.0 for most of the cases.

**Figure 4:**
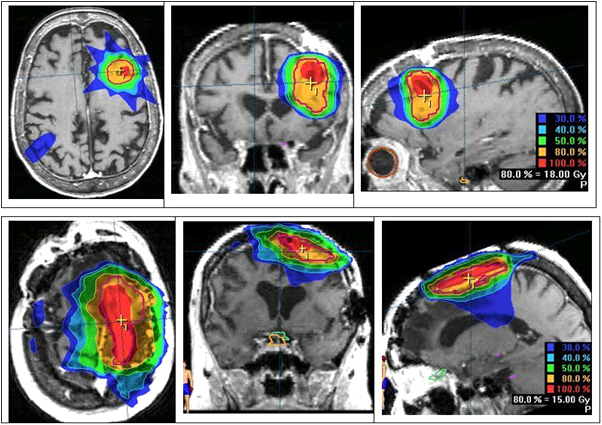
Plot showing axial, coronal and sagittal views of dose distributions of cases 9 (top row) and 11 (bottom row). The corresponding UDI scores are 76 and 573 for cases 9 and 11, respectively.

**Figure 5:**
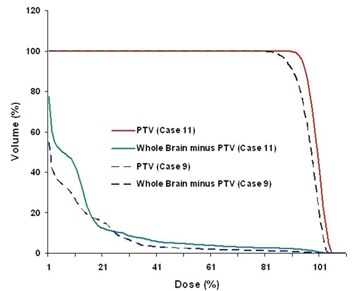
Dose volume histogram (DVH) plots for PTV and Whole Brain minus PTV. The DVH plots for case 9 are shown in dashed lines, and corresponding plots for case 11 are shown in solid lines.

The UDI scores of 21 stereotactic radiosurgery cases evaluated span a wide range. A low UDI score indicates a “good” dosimetry plan, whereas a high score corresponds to a “poor” plan. In order to simplify the analysis, all four components of UDI are equally weighted (i.e, $W_{1}=W_{2}=W_{3}=W_{4}=1.0$). However, in some treatment cases unequal weighting of the UDI components may be more appropriate. The lowest score (corresponding to minimum deviation from an ideal dosimetry plan, UDI=1) is 59, whereas the highest score (corresponding to maximum deviation from an ideal dosimetry plan) is 573 (see Table 1). A histogram plot of the UDI scores is shown in Fig. 2. The UDI score of a dosimetry plan is a single number that gives its overall evaluation in terms of dose coverage, dose conformity, dose homogeneity, as well as dose gradient. This type of scoring is very useful for a consistent quantitative evaluation of a dosimetry plan. In cases where multiple (2 to 3) dosimetry plans are generated, UDI scoring can be an efficient and non‐subjective method for ascertaining which plan is best. In some situations it may also be necessary to score each of the four UDI components separately. A corresponding histogram plot of all four UDI components for the 21 radiosurgery cases is shown in Fig. 3. Dose conformity has the highest value and the wider range of values. In other words, the dose conformity is the more dominant UDI component that determines, to a large extent, how well a dosimetry plan is scored, based on the UDI scoring method. Dose gradient and homogeneity are the second and third dominant UDI components, respectively, and also contribute significantly to the composite UDI score. The dose coverage component, however, does not contribute significantly to the overall UDI score, as indicated in Fig. 3. This is because, for the radiosurgery cases, full target dose coverage is given a high priority in the dosimetry planning. As a result the dose coverage component of UDI is expected to be approximately 1.0. It is important to note that for other modalities such as IMRT the dose coverage component would be more variable, and therefore contribute more to the overall UDI score. One way to demonstrate the effectiveness of UDI scoring method is by associating UDI scores to corresponding dose distributions. Two contrasting examples are shown in Fig. 4. The top panel is taken from case number 9 with a UDI score of 76, whereas the bottom panel is from case number 11 with UDI score of 573. The dose distributions in case 9 show better dose conformity, dose fall‐off, and homogeneity compared to the distributions in case 11. This is consistent with their UDI scores. Corresponding dose volume histogram (DVH) plots for cases 9 and 11 are shown in Fig. 5. DVH plots of the PTV and other contoured critical structures are useful for evaluating the dose coverage to the target, as well as the dose exposure of the organs at risk. Evaluation of the DVH plots of the PTV for cases 9 and 11 show that in both cases the PTV receives excellent dose coverage (i.e., approximately 100% of the target volumes get the prescribed dose which is defined at the 80% isodose line in both cases). However, a DVH plot of the PTV alone is insufficient to ascertain any useful information about dose conformity (i.e., how well the prescribed dose is confined to the intended target volume). However, if we evaluate the dose exposure to normal brain tissue (i.e., whole brain minus PTV), it is apparent that in case 11 a significantly higher volume of normal brain tissue is exposed to higher dose compared to case 9 (see the DVH plot for whole brain minus PTV). This would translate into higher normal tissue complication probability (NTCP) for case 11 compared to case 9, and is consistent with their UDI scores. This further underscores the significance of the dose conformity component of the UDI scoring system.

## IV. DISCUSSION

The UDI scoring method presented has been demonstrated as a tool for efficient and non‐subjective evaluation of a dosimetry plan, as well as for quantitative comparison of multiple plans. As more and more radiation oncology facilities deploy multiple treatment planning systems, it may become necessary to compare one TPS against another. Typically this is done based on side‐by‐side comparison of dose volume histograms and dose distribution obtained from each TPS. The other approach will be to export the dosimetry plans from each TPS to a common platform that can be utilized for more quantitative comparison (which can be quite challenging). The UDI scoring system is a useful tool for evaluation of dosimetry plans obtained from different TPS. Furthermore, the UDI method can also be useful for establishing a benchmark or standard on which dosimetry plans can be ranked.

### A. UDI classification of dosimetry plans

**Figure 6:**
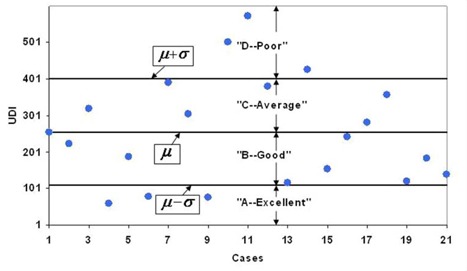
A plot UDI ranking system for cranial radiosurgery plans based on mean (μ) and standard deviation (σ) values obtained for 21 cases. The solid circles show the actual UDI scores. The lines indicate the defined boundaries of the four classification groups. The treatment plans are ranked as “Excellent (A)”, “Good (B)”, “Average (C)”, and “Poor (D)”.

Of the 21 radiosurgery cases presented, the mean UDI score, μ=255, and the standard deviation, σ=144. Four classification groups can be constructed based on the mean and standard deviation, such that UDI scores greater than (μ+σ) are classified as “poor”, whereas scores ranging from (μ) to (μ+ σ) are classified as “average”. UDI scores ranging from (μ‐σ) to (μ) are classified are “good”, and scores less than (μ ‐ σ) are classified as “excellent”. A plot of the ranking system established for cranial stereotactic radiosurgery is shown in Fig.6. Of the 21 cases evaluated, three treatment plans are classified as “excellent”, nine as “good”, six as “average”, and three as “poor”. An example of treatment plan classification using the UDI ranking system is given in Table 2. It is important to emphasize that the ranking system presented in Table 2 is based on UDI data obtained for stereotactic cranial radiosurgery, and therefore is more suitable for such cases. Similar tables should be constructed for ranking dosimetry plans of other treatment modalities, for example, one table for prostate IMRT plans, and another table for head and neck IMRT plans, etc. Furthermore, a ranking table generated by one institution may not be applicable for another institution. It is also important to note that the results presented above are based on a retrospective evaluation of 21 radiosurgery cases treated over a period of a couple of years. The dosimetry plans utilized were determined to be clinically most suitable for treatment. Although some plans rank poorly on the UDI scale, they may very well be the best plans achievable physically for those particular cases based on their target shapes and locations.

**Table 2:** An example of treatment plan classification based on the UDI ranking system. The numbers in braces correspond to cases from the 21 radiosurgery treatment plans evaluated in the study. The mean UDI score, μ=255, and standard deviation, σ<144.

*Plan Classification*	*D (Poor)*	*C (Average)*	*B (Good)*	*A (Excellent)*
UDI Range	UDI≥μ+σ	μ+σ>UDI≥μ	μ>UDI≥μ−σ	UDI<μ−σ
Cases	{10,11,14}	{3,7,8,12,17,18}	{1,2,5,13,15,16,19,20,21}	{4,6,9}

### B. UDI variance with planning technique

It is also desirable to have a tool (other than DVH) that can be utilized during the planning process as a quick evaluation guide that will help the planner judge how well a dosimetry plan is meeting the dose coverage, conformity, homogeneity or dose gradient goals. UDI can be used to select the best plan from multiple competing plans, for the same case. For instance, is there a big difference using 7 beams versus 9 beams? Or is there a big difference using static coplanar versus dynamic arcs? Such questions can be answered based on UDI scoring. An illustration of UDI variance as a function of number of treatment beams is presented in Table 3. The planning targets and structures from cases 9 and 11 are utilized. In order to facilitate a systematic analysis the treatment beams utilized for these calculations are coplanar and equally spaced, and equally weighted. For case 9 the mean UDI score, μ=62, and standard deviation, σ=6.5. For case 11 the corresponding values are μ=457, and σ=9.9. In general, a UDI variance of 5 to 10 (higher variance values for targets with more irregular shape) is considered significant.

**Table 3:** Illustration of UDI variance as a function of number of treatment beams for cases 9 and 11. For systematic comparison the treatment beams are equally spaced and coplanar, and equally weighted. Each set of beams includes 0° gantry angle. For case 9 the mean UDI score, μ=62, and standard deviation, σ=6.5. For case 11 the corresponding values are μ=457, and σ=9.9.

	*UDI*	
*Number of Coplanar Beams*	*Case 9*	*Case 11*
5	60	469
6	72	458
7	62	460
8	56	448
9	67	444
10	55	465

### C. Comparing UDI to other scoring systems

Whereas a number of indices for scoring dosimetry plans have been proposed,^1^, ^11^ it is difficult to get a good sense as to which system is better or more useful. The Conformity/Gradient Index (CGI) of Wagner et al.^(1)^ has been widely used and quoted in the literature.^11^, ^15^ The CGI index averages the components of dose conformity and dose gradient. This can lead to ambiguous scoring due to the effect of averaging. The Radiation Therapy Oncology Group (RTOG) have also suggested a quality assurance guideline for radiosurgery treatment plans, which include three separate indices of dose coverage, conformity, and homogeneity.^(16)^ The unified dosimetry index presented here, is the only system that incorporates all four dosimetry components of dose coverage, conformity, homogeneity and dose gradient into a single overall score. The UDI method compounds the contributions from each component, instead of averaging as is the case in the CGI method of Wagner et al.,^(1)^ thereby avoiding ambiguous scoring.

A limitation of the UDI system is that it is not easily utilized for scoring IMRT cases in which multiple target dose coverage objectives are specified. Furthermore, although dose gradient component is included, there is no direct accounting of critical structure dose in the UDI scoring system. This point should be taken into consideration when analyzing UDI scores, since in some clinical situations minimizing critical structure dose could be an overriding priority.

## V. CONCLUSION

The UDI method unifies four dosimetry objectives of dose coverage, conformity, homogeneity, and dose gradient, into one simple equation that is easily utilized for calculating a figure of merit that quantifies the overall quality of a dosimetry plan. We propose a technique for creating a ranking table that can be used for plan evaluation and comparison between multiple plans. Under this system treatment plans can be classified as “excellent”, “good”, “average”, or “poor”. A separate ranking table would be needed for each treatment modality (e.g., cranial radiosurgery, IMRT for prostate and head and neck cases, etc).

## References

[1] Wagner TH , Bova FJ , et al. A simple and reliable index for scoring rival stereotactic radiosurgery plans. Int J Radiat Oncol Biol Phys. 2003;57(4):1141–1149. 14575847 10.1016/s0360-3016(03)01563-3

[2] Lomax NJ , Scheib SG . Quantifying the degree of conformity in radiosurgery treatment planning. Int J Radiat Oncol Biol Phys. 2003;55(5):1409–1419. 12654454 10.1016/s0360-3016(02)04599-6

[3] Bova FJ , Meeks SA , Friedman WA , et al. Stereotactic plan evaluation tool “the UF index”. Int J Radiat Oncol Biol Phys. 1999;45(3):188.

[4] van't Riet A , Mak AC , Moerland MA , et al. A conformation number to quantify the degree of conformality in brachytherapy and external beam irradiation: application to the prostate. Int J Radiat Oncol Biol Phys. 1997;37(3):731–736. 9112473 10.1016/s0360-3016(96)00601-3

[5] Baltas D , Kolotas C , Geramani K , et al. A conformal index (COIN) to evaluate implant quality and dose specification in brachytherapy. Int J Radiat Oncol Biol Phys. 1998;40(2):515–524. 9457842 10.1016/s0360-3016(97)00732-3

[6] Oozeer R , Chauvet B , Garcia R , et al. Dosimetric evaluation of conformal radiotherapy: Conformity factor. Cancer Radiother. 2000;4(3):207–216. 10897764 10.1016/s1278-3218(00)89096-4

[7] Knoos T , Kristensen I , Nilsson P . Volumetric and dosimetric evaluation of radiation treatment plans: Radiation conformity index. Int J Radiat Oncol Biol Phys 1998;42(5): 1169–1176. 9869245 10.1016/s0360-3016(98)00239-9

[8] Paddick I . A simple scoring ratio to index the conformity of radiosurgical treatment plans. Technical note. J Neurosurg. 2000;93(3 Suppl):219–222. 11143252 10.3171/jns.2000.93.supplement

[9] Leung LH , Chua DT , Wu PM . A new tool for dose conformity evaluation of radiosurgery treatment plans. Int J Radiat Oncol Biol Phys. 1999;45(1):233–241. 10477028 10.1016/s0360-3016(99)00175-3

[10] Wu VWC , Kwong DLW , Sham JST . Target dose conformity in 3‐dimensional conformal radiotherapy and intensity modulated radiotherapy. Radiother Oncol. 2004;71(2):201–206. 15110454 10.1016/j.radonc.2004.03.004

[11] Feuvret L , Noel G , Mazeron JJ , et al. Conformity index: A review. Int J Radiat Oncol Biol Phys. 2006;64(2):333–342. 16414369 10.1016/j.ijrobp.2005.09.028

[12] Chang SD , Gibbs IC , Sakamoto GT , et al. Staged stereotactic irradiation for acoustic neuroma. Neurosurgery. 2005;56(6):1254–1261. 15918941 10.1227/01.neu.0000159650.79833.2b

[13] International Commission on Radiation Units and Measurements (ICRU) . Prescribing, recording and reporting photon beam therapy. ICRU Report 50. Bethesda (MD):ICRU;1993.

[14] International Commission on Radiation Units and Measurements (ICRU) . Prescribing, recording and reporting photon beam therapy. ICRU Report 62. Bethesda, (MD): ICRU;1999.

[15] Han C , Liu A , Schultheiss TE , et al. Dosimetric comparisons of helical tomotherapy treatment plans and step‐and‐shoot intensity‐modulated radiosurgery treatment plans in intracranial stereotactic radiosurgery. Int J Radiat Oncol Biol Phys. 2006;65(2):608–616. 16690442 10.1016/j.ijrobp.2006.01.045

[16] Shaw E , Kline R , Gillin M , et al. Radiation Therapy Oncology Group: Radiosurgery quality assurance guidelines. Int J Radiat Oncol Biol Phys. 1993;27(5):1231–1239. 8262852 10.1016/0360-3016(93)90548-a

